# Supplementary figures and images for: CLASPP: A unified model for predicting post-translational modifications
Source: bioRxiv. 2026 Jun 6:2026.06.04.729962. Preprint. [Version 1] doi: 10.64898/2026.06.04.729962 (PMC13252087; doi:10.64898/2026.06.04.729962)

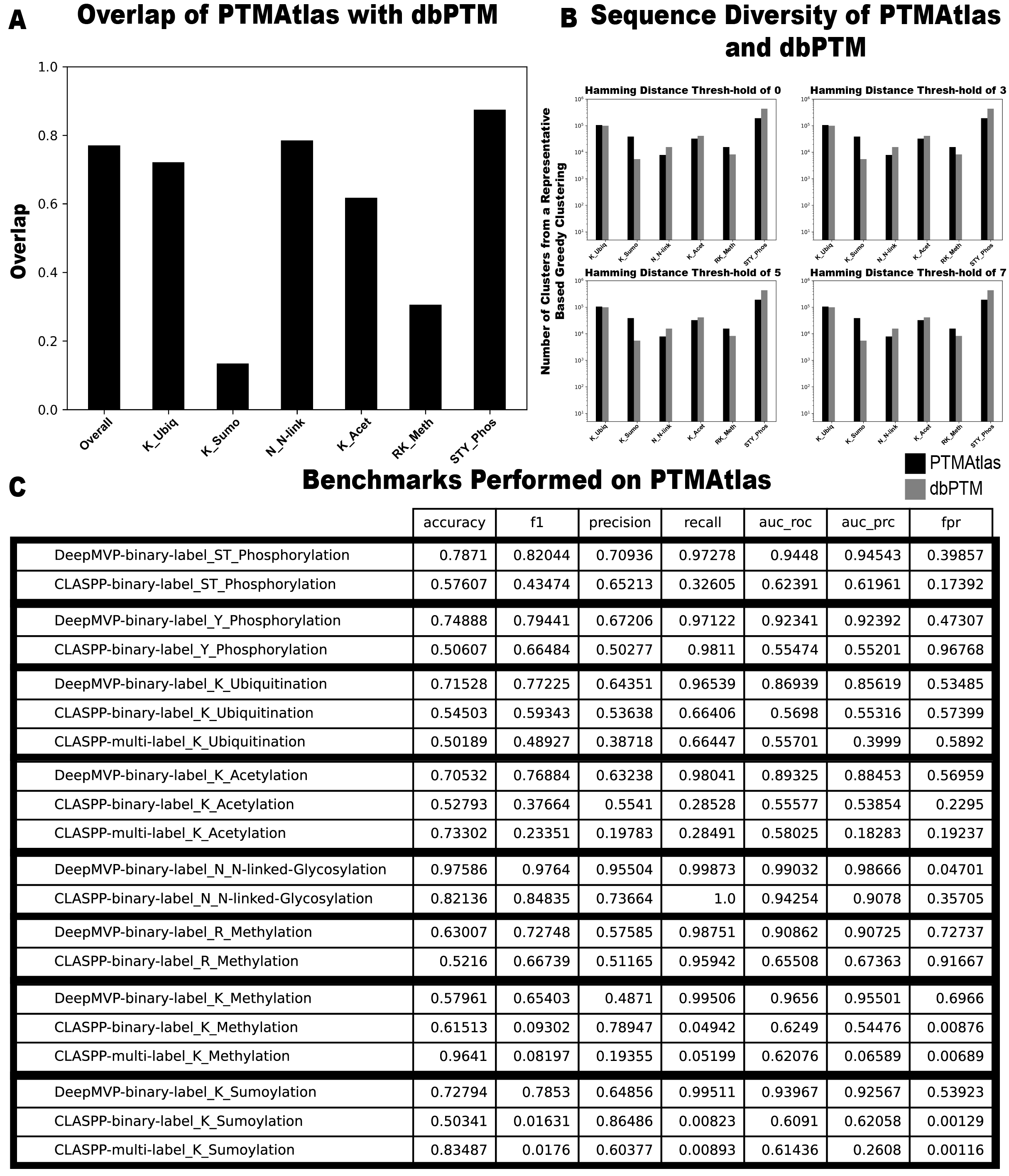

Supplement: Supplement 5 — S5 Fig. Comparison of PTMAtlas and dbPTM (A) Probability of PTMAtlas overlapping with dbPTM of positive 21-mer sequences per PTM type [9, 21]. (B) Measure of sequence diversity per PTM type for PTMAtlas and dbPTM. The metric of sequence diversity was measured by the log scaled number of clusters from a representative greedy clustering of the positive 21-mer sequences. Different thresholds were systematically tested. (C) Benchmarks of DeepMVP [21] and CLASPP ran on PTMAtlas’s testing set. Binary-label benchmarks refer to using the exact test data supplied by PTMAtlas. Multi-label benchmarks are the altered test sets where the positive data points from adjacent PTM types are added to the negative class of interest so long as the 21-mer input does not exist in the positive class of interest and they share the central modified residue type. [file media-5.zip › S5Fig.png]

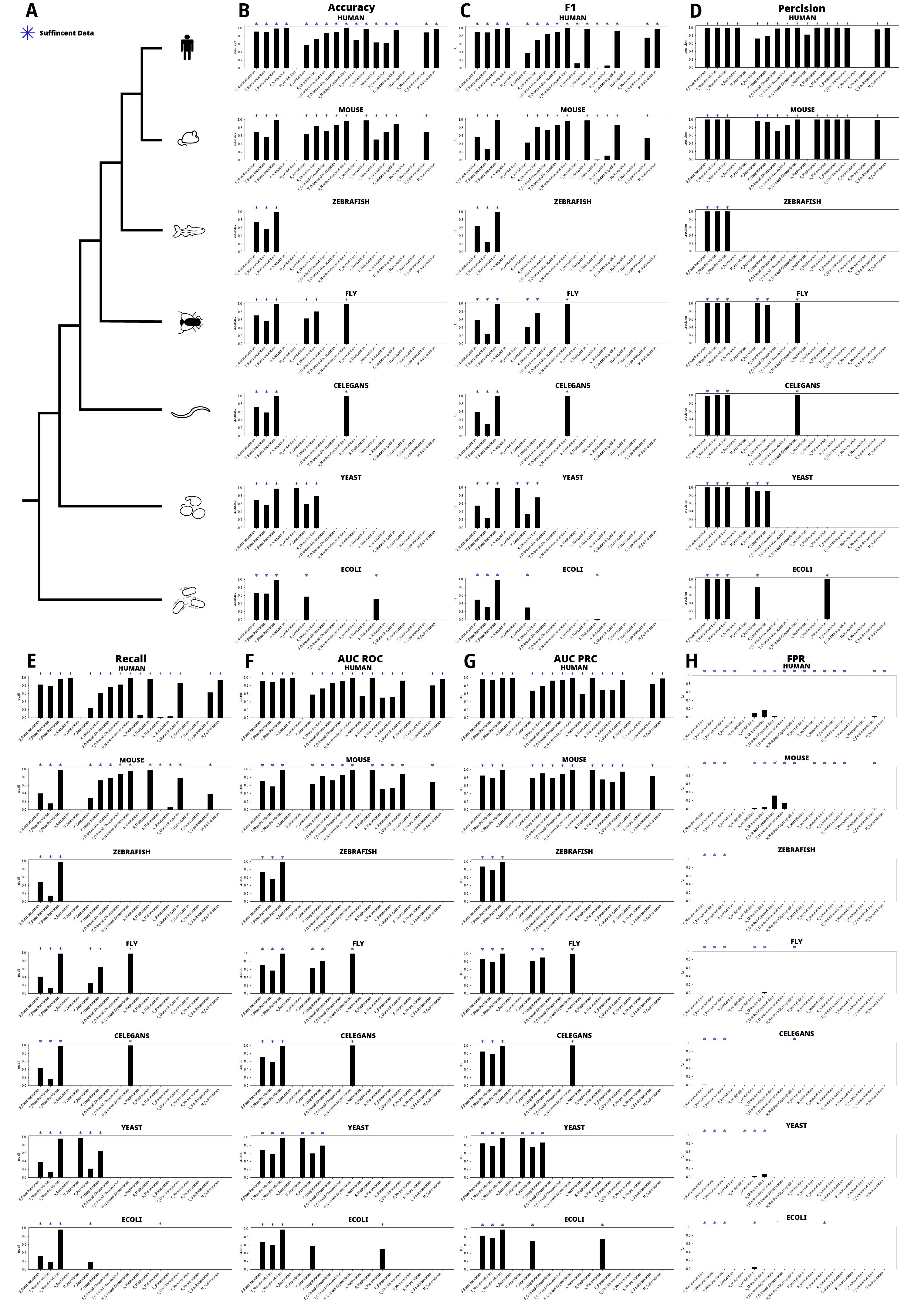

Supplement: Supplement 6 — S6 Fig. Comprehensive out-of-distribution benchmark. (A) Demonstrates relative evolutionary distance and this metric is a way to measure the degree of out-of-distribution for a given species-specific benchmark. Human refers to H. sapiens, Mouse refers to M. musculus, Zebrafish refers to D. rerio, Fly refers to D. melanogaster, Celegans refers to C, elegans, Yeast refers to S. cerevisiae, and Ecoli refers to E. coli. (B) This out-of-distribution benchmark is for the metric accuracy. (C) This out-of-distribution benchmark is for the metric F1. (D) This out-of-distribution benchmark is for the metric precision. (E) This out-of-distribution benchmark is for the metric recall. (F) This out-of-distribution benchmark is for the metric AUC-ROC. (G) This out-of-distribution benchmark is for the metric AUC-PRC. (H) This out-of-distribution benchmark is for the metric FPR. (C-H) The benchmark only contributes to the Fig 5c if a blue star is present representing sufficient data. [file media-6.zip › S6Fig.png]
